# Dysregulated immune proteins in plasma in the UK Biobank predict multiple myeloma 12 years before clinical diagnosis

**DOI:** 10.1182/bloodadvances.2025016120

**Published:** 2025-05-09

**Authors:** Joshua Fieggen, Anshul Thakur, Christopher C. Butler, Karthik Ramasamy, Anjan Thakurta, David A. Clifton, Lei Clifton

**Affiliations:** 1 Computational Health Informatics Lab, Institute of Biomedical Engineering, Department of Engineering Sciences, University of Oxford, Oxford, United Kingdom; 2 Infection, Respiratory, and Acute Care, Nuffield Department of Primary Care Health Sciences, University of Oxford, Oxford, United Kingdom; 3 Oxford Translational Myeloma Centre, Nuffield Department of Orthopaedics, Rheumatology and Musculoskeletal Sciences, University of Oxford, Oxford, United Kingdom; 4 Department of Haematology, Oxford University Hospitals National Health Service Foundation Trust and Oxford Translational Myeloma Centre, University of Oxford, Oxford, United Kingdom; 5 Mathematical, Physical, and Life Sciences Division, Oxford Suzhou Centre for Advanced Research, University of Oxford, Suzhou, China; 6 Applied Digital Health, Nuffield Department of Primary Care Health Sciences, University of Oxford, Oxford, United Kingdom

**TO THE EDITOR:**

Multiple myeloma (myeloma) represents a significant clinical challenge because of its symptom burden at diagnosis, often a consequence of delayed presentations.^1^ There are only a few specific risk factors for myeloma, and diagnosis is often only made following complications, such as anemia, bone lesions, renal failure, and immune dysregulation.^2^ Although myeloma remains incurable, early diagnosis is critical to improving outcomes.^3^

Proteomics has emerged as a pivotal tool in cancer research, offering insights into the molecular basis of various malignancies.^4^ Early myeloma and its precursor disease states present with a high quantity of immunoglobin paraprotein in the blood as a key marker of disease.^2^ Indeed, mass spectrometry–based serum proteomics have been shown to be able to effectively differentiate between cases of monoclonal gammopathy of uncertain significance (MGUS) and healthy controls.^5^ In addition, protein levels of albumin, β2 microglobulin, and lactate dehydrogenase are typically assessed to risk-stratify patients.^6^ However, a proteomics-based diagnostic test has not been developed for myeloma. It is also plausible that plasma from healthy individuals may contain proteins from organs and/or the immune system that serve as biomarkers of physiological dysregulation that precedes the onset of disease. The availability of Olink plasma proteomic data^7^ of 2932 unique proteins from 54 219 healthy participants with a long-term clinical follow-up in the UK Biobank^8^ enabled us to explore this possibility.

Our study population (supplemental Figure 1) includes all UK Biobank participants with available baseline plasma proteomics data and excluded prevalent myeloma cases (those with existing diagnoses at baseline). The disease outcome was defined as incident myeloma cases (diagnoses of myeloma after the baseline date) that were identified through linked cancer registry, death registry, and in-patient hospital records. All Olink data quality control steps have been described previously,^7^ including scaling and normalizing the data around a median of zero to account for potential intra- and interbatch variation. Thus, the proteomics data represent the relative rather than absolute plasma protein concentrations. To identify the top 10 proteins that are predictive of myeloma onset, we employed a machine learning–based feature selection pipeline (Figure 1A) using an extreme gradient boosting (XGBoost) algorithm with a Cox loss function and Shapley additive explanations (SHAP).^9^ These were then used with and compared against the best available clinical variables known to predict myeloma in the general UK population^10^ (supplemental Method 1). To do this, 3 Cox models (1 using clinical variables, 1 with proteomic biomarkers, and the third combined model that incorporated both) were developed with 80% of the data and tested on the remaining 20%, and performance was assessed using time-dependent receiver operating characteristic curves and concordance (C) indexes. The detailed statistical methods are described in supplemental Method 2.

**Figure 1.**
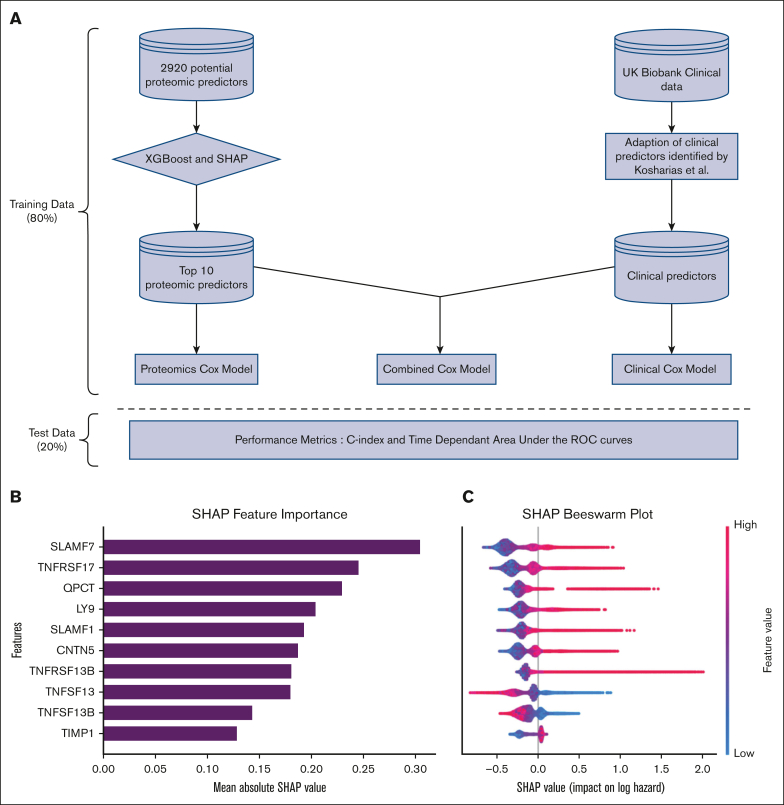
**Model development pipeline and top proteomic machine learning model features.** (A) Outline of the pipeline used to predict myeloma by integrating proteomic and clinical data from the UK Biobank (UKB). Starting with 2920 potential proteomic predictors, a tree-based XGBoost algorithm, combined with SHAP values, was employed to rank and identify the top 10 predictors. These were then used to develop a proteomics Cox model. Clinical predictors, including age, sex, symptoms, and hematologic parameters were used to develop a clinical Cox model. Finally, the top proteomic and clinical predictors were combined to create a combined Cox model. All models were evaluated on the test data set with performance assessed using the C index and time-dependent area under the receiver operating characteristic curve. This pipeline demonstrates how advanced machine learning can be combined with traditional modelling to enhance the prediction of myeloma. (B) A bar plot of the mean absolute SHAP values for the top 10 features. In the context of a model with a Cox-loss function, a SHAP value represents the marginal contribution of each feature to the log-relative hazard (ie, risk score) from baseline for an individual. This panel provides a summary of the average of all individual contributions to the model’s predictions. The features are ranked with higher values indicating greater importance in influencing the model’s output, thereby providing a comparison of which proteomic markers are most critical in ranking myeloma hazard. (C) A scatterplot (beeswarm plot) in which each dot represents an individual data point in the data set. The points are distributed horizontally along the x-axis according to their SHAP value. Where there is a high density of similar SHAP values, points are stacked vertically. The color of the dots reflects the feature value with red indicating high feature values and blue indicating low feature values. The plot provides a granular view of how each feature contributes to the prediction at an individual level. It shows the distribution of SHAP values for each feature, revealing how consistently (or inconsistently) a feature affects the model’s output across different data points. Features with a wide range of SHAP values indicate a strong but varied impact on the model’s predictions, whereas a narrow range suggests a more uniform influence.

Incident myeloma was diagnosed in 174 (0.3%) of the cohort participants (supplemental Table 1) with a median time to diagnosis of 7.2 years and a whole-cohort median follow-up of 13.2 years. Participants diagnosed with myeloma were older and more likely to be male. Further baseline clinical characteristics are shown in supplemental Table 1.

The top 10 of the 2920 features from the optimized XGBoost model, as ranked by the mean absolute SHAP value, are shown in Figure 1B, and supplemental Table 2 describes the function,^11^ location,^12^ single-cell expression,^12^ and their role as targets in myeloma therapeutics.^13^ It is notable that at least 7 of these proteins have known biologic functions in lymphoid cells. This includes 3 signaling lymphocytic activation molecule (SLAM) family receptors, multiple of which are either current or potential targets of antimyeloma immunotherapies.^14^ Targets also identified by the algorithm were the interacting ligands and receptors of B-cell activating factor (BAFF), a proliferation-inducing ligand (APRIL), B-cell maturation antigen (BCMA), and transmembrane activator and calcium modulating ligand interactor (TACI), which have known relevance to myeloma pathophysiology.^15^^,^^16^ Although glutaminyl-peptide cylotransferase (QPCT) and contactin 5 (CNTN5) are not currently known to have any clear function related to B-cell biology or myeloma development, both have been noted to be upregulated in the plasma cells of some patients with myeloma at the single-cell level.^17^^,^^18^ TIMP metallopeptidase inhibitor 1 (TIMP1) is a nonspecific metalloprotease inhibitor involved in innate immunity.

The individual SHAP values (Figure 1C) show the marginal contribution of each protein to the log-relative hazard (analogous to risk score) from baseline for the individuals. The top 7 proteins identified showed a positive association in that high relative protein concentrations were associated with a higher predicted risk for future myeloma. Curiously, APRIL/TNF superfamily member 13 (TNFSF13) and BAFF/TNFSF13B showed the opposite effect in that higher relative concentrations were associated with lower risk scores. This seems to be in contrast with previous literature that suggested that APRIL and BAFF are potential markers of myeloma disease activity;^19^ however, given that these proteins are involved in normal B-cell functioning, this may suggest their complex role in the immune dysregulation that precedes the clonal proliferation of malignant plasma cells. When SHAP plots were used to explore interactions (supplemental Figure 2), there was a clearer interaction pattern identified between TACI and APRIL than between TACI and BAFF despite both these ligands being known to bind TACI.

The distributions of the relative concentrations of the top protein predictors stratified by incident myeloma status are shown in Figure 2A. We used these proteins to construct our first Cox model (red, Figure 2B) in which multiple proteomics markers were statistically significant predictors of myeloma. Notably, SLAMF7, TNFRSF17 (BCMA), QPCT, SLAMF1, and CNTN5 were associated with higher, statistically significant hazard ratios. Conversely, BAFF (TNFSF13B) had a protective effect in accordance with the SHAP findings. In the clinical model (black, Figure 2B), older age, male sex, and lower hemoglobin were associated with higher risk. In the combined model (gray, Figure 2B), age remained a significant predictor, whereas, notably, sex lost statistical significance, potentially suggesting that variance clinically attributable to sex is captured by the proteomics features. The proteomic markers remained significant and almost identical in magnitude in the combined model as in the proteomics only model underscoring their robust association with disease. In a sensitivity analysis that excluded cases diagnosed within 5 years (supplemental Figure 3), the noted proteomic associations largely persisted.

**Figure 2.**
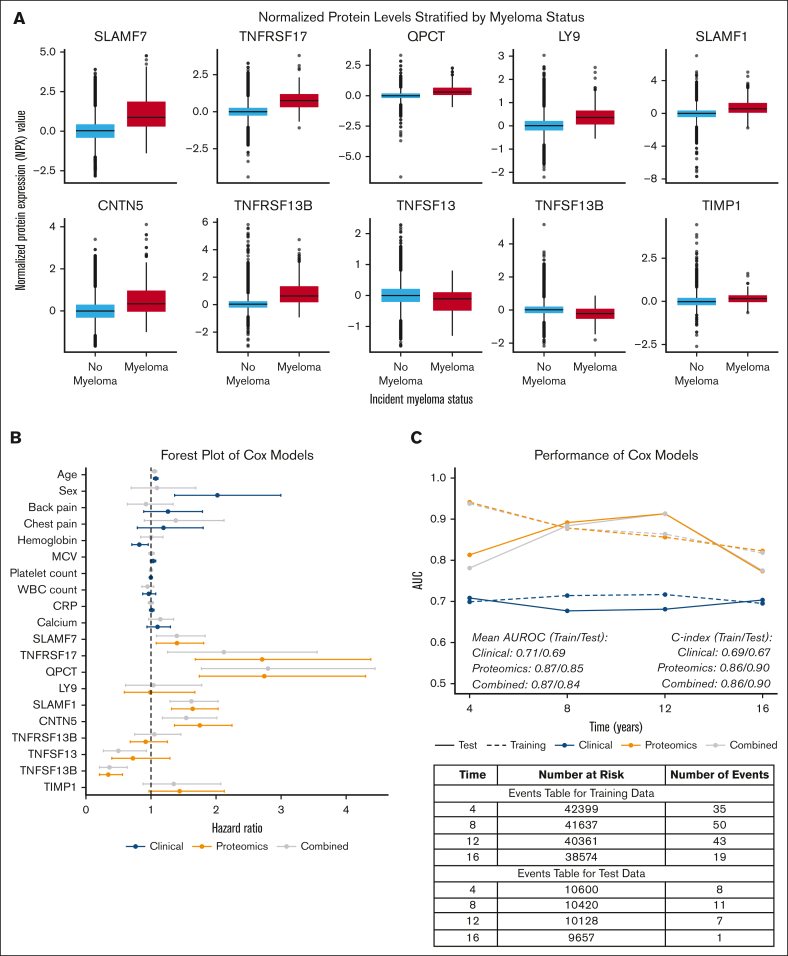
**Associations between the proteomic and clinical features for incident myeloma.** (A) Box plots of the normalized protein expression (NPX) values of each of the top 10 proteomic features at baseline (enrolment into UK Biobank [UKB]), stratified by incident myeloma status. The plots are arranged in order of SHAP importance. The box plot lines represent the median NPX value, edges represent the first and third quartiles, and whiskers show 1.5× the interquartile range with dots as outliers outside this range. To account for the intra- and interbatch variability, the Olink data were scaled and normalized around a median of zero; thus, half of the data have negative values, and the data represent relative rather than absolute protein concentrations. (B) A forest plot of the hazard ratios estimated from the 3 Cox models that were developed. The proteomics model is shown in orange, the clinical model in blue, and the combined model in gray. The hazard ratio point estimates are represented by the dot and the 95% confidence intervals are represented by the whiskers. (C) A comparison of the performance of the 3 Cox models on the training and held-out test data sets. The line plot displays the time-dependent area under the receiver operator characteristic curves (AUROCs) for each of the 3 models at years 4, 8, 12, and 16 since enrolment in the UKB with the mean AUROCs and overall C indexes in the training and test data summarized below. The table below presents the number of participants in the training and test data sets at risk at years 4, 8, 12, and 16, respectively, and the number events (myeloma diagnoses) that occurred between years 0 to 4, 4 to 8, 8 to 12, and 12 to 16.

The clinical model had the lowest performance on both the training and test data with a C index of 0.69. In contrast, the proteomics and combined models performed very similar and outperformed the clinical model substantially. Both had C indexes of 0.86 and 0.90 in the training and test data, respectively. The model performance improved in the test data for both the proteomics and combined models at each 4-year time interval until 12 years of follow-up (Figure 2C). These results suggest that plasma may contain biomarkers that long precede disease defining events.

This analysis shows that a hypothesis-free and data-driven approach may capture patterns that reflect biologic B-cell/immune dysregulation that precedes the onset of clinical disease in myeloma. In the context of recent literature that potentially supports population MGUS screening,^20^ a better understanding of the mechanisms that lead to progression to myeloma is increasingly important. In addition, a recent study demonstrated that matrix-assisted laser desorption/ionization time of flight mass spectrometry of the serum proteome can identify MGUS, supporting the case for plasma protein–based methods across different detection modalities.^5^

An important limitation of this study in this respect is the inability to comprehensively describe participant MGUS status or baseline paraprotein concentrations. To attempt to understand what impact this may make, we refitted our Cox models and excluded all prevalent and incident cases of MGUS (supplemental Figure 4). This analysis showed that proteomic associations were largely unaffected by the removal of all MGUS cases. Given that it has recently been shown that MGUS that has been detected through screening and incidental finding have a similar progression risk,^20^ this finding gives us more confidence that the identified proteomic associations are important, independent of the underlying MGUS status.

The increasing attention on MGUS highlights the need for further research to understand how these markers change dynamically as individuals move from a healthy baseline through various precursor states and into clinical disease. In addition, this work should be developed further to explore whether the predictive performance can be maintained with fewer proteins or be improved by considering interactions with more commonly measured clinical markers, such as total protein and albumin. Orthogonal biologic approaches need to be considered to identify and understand the source and biologic implications of the proteins identified and to identify viable clinical assays for the identified proteins. Finally, in future, it will be key to distinguish between and optimize models for time windows relevant to specific clinical questions and to benchmark against alternative methods used in liquid biopsies.

**Conflict-of-interest disclosure:** The authors declare no competing financial interests.

## Supplementary Material

Supplemental Methods, Tables, Figures, and References
